# A VIGS screen identifies immunity in the Arabidopsis Pla‐1 accession to viruses in two different genera of the Geminiviridae

**DOI:** 10.1111/tpj.13716

**Published:** 2017-10-24

**Authors:** Maria Ines Reyes, Miguel A. Flores‐Vergara, Orlene Guerra‐Peraza, Cyprian Rajabu, Jigar Desai, Yokiko H. Hiromoto‐Ruiz, Joseph Ndunguru, Linda Hanley‐Bowdoin, Susanne Kjemtrup, Jose T. Ascencio‐Ibáñez, Dominique Robertson

**Affiliations:** ^1^ Department of Plant and Microbial Biology North Carolina State University Raleigh NC USA; ^2^ Paradigm Genetics Research Triangle Park NC USA; ^3^ Mikocheni Agricultural Research Institute Dar es Salaam Tanzania; ^4^ Department of Molecular and Structural Biochemistry North Carolina State University Raleigh NC USA; ^5^ Present address: Citrus Research and Education Center University of Florida Lake Alfred FL 33850 USA; ^6^ Present address: Department of Plant and Microbial Biology North Carolina State University Raleigh NC USA

**Keywords:** Arabidopsis, BCTV, CaLCuV, geminivirus, *gip‐1*, immunity, Pla‐1, VIGS

## Abstract

Geminiviruses are DNA viruses that cause severe crop losses in different parts of the world, and there is a need for genetic sources of resistance to help combat them. Arabidopsis has been used as a source for virus‐resistant genes that derive from alterations in essential host factors. We used a virus‐induced gene silencing (VIGS) vector derived from the geminivirus *Cabbage leaf curl virus* (CaLCuV) to assess natural variation in virus–host interactions in 190 Arabidopsis accessions. Silencing of *CH‐42*, encoding a protein needed to make chlorophyll, was used as a visible marker to discriminate asymptomatic accessions from those showing resistance. There was a wide range in symptom severity and extent of silencing in different accessions, but two correlations could be made. Lines with severe symptoms uniformly lacked extensive VIGS, and lines that showed attenuated symptoms over time (recovery) showed a concomitant increase in the extent of VIGS. One accession, Pla‐1, lacked both symptoms and silencing, and was immune to wild‐type infectious clones corresponding to CaLCuV or *Beet curly top virus* (BCTV), which are classified in different genera in the Geminiviridae. It also showed resistance to the agronomically important *Tomato yellow leaf curl virus* (TYLCV). Quantitative trait locus mapping of a Pla‐1 X Col‐0 F_2_ population was used to detect a major peak on chromosome 1, which is designated *gip‐1* (*geminivirus immunity Pla‐1‐1*). The recessive nature of resistance to CaLCuV and the lack of obvious candidate genes near the *gip‐1* locus suggest that a novel resistance gene(s) confers immunity.

## Significance Statement

We explored virus‐induced gene silencing (VIGS) responses using a geminivirus vector and found surprisingly diverse responses in 190 Arabidopsis accessions. Only the Pla‐1 accession was resistant to VIGS, and to three diverse wild‐type geminiviruses. Mapping studies revealed a novel recessive locus that, once identified, could bolster breeding efforts for resistance against these devastating DNA viruses. Broad‐based immunity is necessary to combat the high rate of geminivirus evolution and the frequent occurrence of mixed infections.

## Introduction

The Geminiviridae is a large family of circular, single‐stranded DNA (ssDNA) plant viruses named for their twinned particles (Hanley‐Bowdoin *et al*., 2013). They are classified into different genera depending on their insect vector, genome structure and host range (Hanley‐Bowdoin *et al*., 2013; Varsani *et al*., 2014). As a group, geminiviruses infect a broad range of crop plants primarily in tropical and subtropical regions of the world (Moffat, 1999). Their incidence and severity have increased over the past 20 years (Mansoor *et al*., 2006; Navas‐Castillo *et al*., 2011), and some resistance strategies used to control them are no longer effective. Breakdown of resistance has been associated with novel disease agents, including ssDNA alphasatellites and betasatellites as well as host‐derived sequences enhancing geminivirus symptoms (Nawaz‐ul‐Rehman and Fauquet, 2009; Ndunguru *et al*., 2016), which are of concern because of their recent emergence and unknown etiology.

Two resistance genes that provide resistance to *Tomato yellow leaf curl virus* (TYLCV), a monopartite member of the Begomovirus genus, were recently identified in tomato. Variations in the first gene, *Ty‐1/Ty‐3*, which encodes a gamma‐type RNA‐dependent RNA polymerase (RDR), were found to be responsible for resistance in two different lines of tomato (Verlaan *et al*., 2013). Resistance most likely involves augmentation of host gene silencing pathways, and has been associated with increased methylation of viral DNA (Butterbach *et al*., 2014). The second resistance gene, *ty‐5*, encodes an altered version of Pelota (*Pelo*), which functions in ribosome recycling following translation (Lapidot *et al*., 2015). This recessive resistance is important because it identified an essential host factor and because it will be difficult for the virus to overcome. Identification of both of these genes has facilitated breeding for geminivirus resistance. However, neither resistance gene prevents the accumulation of viral DNA during infection, which can lead to the development of viral mutations that eventually overcome resistance (Arguello‐Astorga *et al*., 2007; Richter *et al*., 2016). In addition, there is a risk that Ty‐1/Ty‐3 resistance will be overcome because geminiviruses encode anti‐silencing proteins (Raja *et al*., 2010) and occur in mixed infections with pathogenic RNA viruses, such as *Cassava brown streak virus* (Mbanzibwa *et al*., 2009), which also interfere with silencing. Geminivirus‐associated satellites frequently target host gene silencing pathways (Nawaz‐ul‐Rehman and Fauquet, 2009; Hanley‐Bowdoin *et al*., 2013). There is a need for additional sources of resistance to augment the existing repertoire and enhance the possibility of creating durable resistance.

*Arabidopsis thaliana* has served as a model plant for studying virus–host interactions, and numerous genes impacting infection have been identified using mutagenized populations or by screening naturally occurring accessions (Ouibrahim *et al*., 2014). Because Arabidopsis is self‐pollinating, the thousands of accessions collected from around the world function as inbred lines that have adapted to a wide range of environments (Consortium, 2016). These accessions can be used for genome‐wide association studies (GWAS) in addition to quantitative trait locus (QTL) mapping, and provide powerful tools for uncovering the genetic basis of defective virus–host interactions that can lead to resistance (Pagny *et al*., 2012). For example, the potyvirus resistance gene, *eIF4(iso)E*, was first identified in an Arabidopsis mutant (Lellis *et al*., 2002), and then found to correspond to the broad spectrum potyvirus resistance allele (*pvr*) that is widely used for breeding (Ruffel *et al*., 2002; Kang *et al*., 2005). The product of this recessive resistance gene participates in translation initiation, and has been called the ‘weak link’ of potyvirus infection (Robaglia and Caranta, 2006). Three resistance genes that limit *Tobacco etch virus* to inoculated leaves were identified in a mutagenized population of Ler‐1 after the trait was first uncovered in naturally occurring populations (Chisholm *et al*., 2000). Recently, the Cvi‐0 accession was used to identify a variant form of a phosphoglycerate kinase gene that confers potyvirus resistance (Ouibrahim *et al*., 2014) and provided new insights into the involvement of metabolic enzymes in virus–host interactions.

Several years ago, we initiated a screen of Arabidopsis accessions to better understand geminivirus–plant interactions. We were interested in using Arabidopsis to study virus‐induced gene silencing (VIGS). VIGS takes advantage of a major plant defense pathway, post‐transcriptional gene silencing (PTGS), which results in degradation of the aberrant RNA associated with viral infection (Waterhouse *et al*., 2001). When a host gene fragment is inserted into the virus, mRNA from the host gene is also degraded. Previously (Turnage *et al*., 2002), we showed that Col‐0 plants inoculated with a VIGS vector derived from the geminivirus *Cabbage leaf curl virus* (CaLCuV) carrying a fragment of the *CH‐42* gene (At4G18480; a.k.a. *Chlorina‐42*,* CHLI‐1*), which encodes magnesium chelatase subunit I, developed yellow‐white areas due to chlorophyll loss. However, viral symptoms were too severe for its use as a VIGS vector. We screened 190 accessions to identify lines with attenuated symptoms and accidentally identified one line, Pla‐1, that showed durable and complete resistance to CaLCuV even when wild‐type virus was used for infection. Because of the potential benefits of a natural source of immunity to geminiviruses, we chose to focus on Pla‐1. This paper reports results of the VIGS screen and our initial characterization of Pla‐1 immunity.

## Results

### Diverse responses to the VIGS vector

We conducted a screen of Arabidopsis accessions to assess natural variation in response to a geminivirus VIGS vector. The original motivation was to find lines with reduced symptoms, but we were also interested in resistance. Preliminary experiments showed that VIGS in Col‐0 was more extensive under short‐day conditions (8 h light/16 h dark), which were used for the screen. To standardize results, Col‐0 was included in each experiment as an internal control.

Initially, the CaLCuV vector with or without a non‐homologous *Luciferase* fragment was used simply to assess symptoms. When it was realized that the extent of VIGS also varied among accessions, CaLCuV carrying a *CH‐42* fragment was used, and both symptoms and the extent of silencing were scored. Results from the first experiments are included because each of the 26 accessions tested showed symptoms (Figure S1; Table S1), indicating that they were not resistant to CaLCuV.

**Figure 1:**
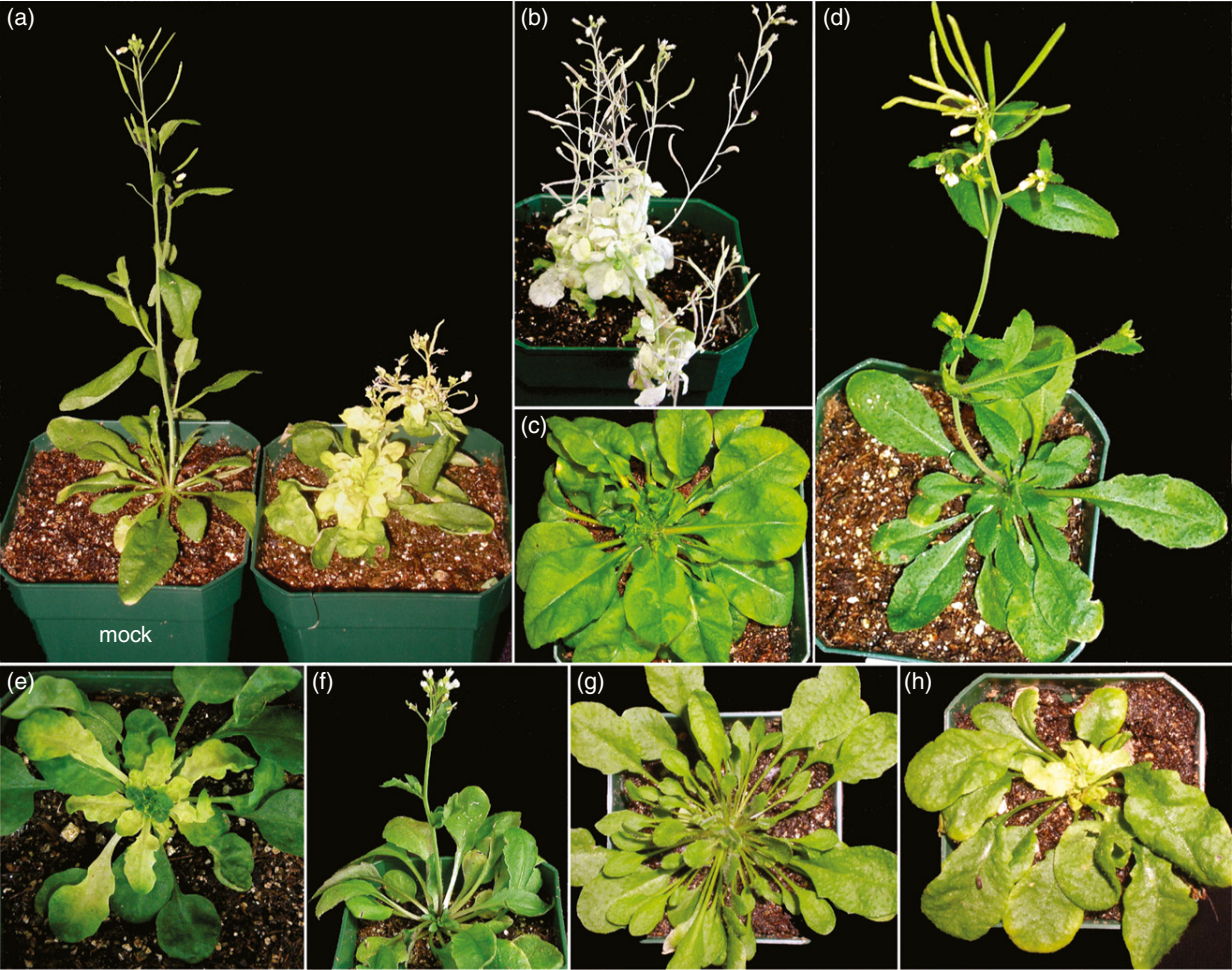
Response of different Arabidopsis accessions to the CaLCuVA:CH‐42 virus‐induced gene silencing (VIGS) vector (a–f) or wild‐type CaLCuV (g and h). (a) Hi‐0, a Class A accession, at 29 dpi. Hi‐0 on the left was not inoculated. (b) Kil‐0, a Class B accession, at 54 dpi. (c) Gr‐1, a Class C accession, at 25 dpi. (d) Di‐0, a Class D accession, at 27 dpi. (e) Li‐2:1, a Class D accession that shows recovery at 29 dpi (f) and 60 dpi. (g) Pla‐1, which never showed silencing or symptoms, at 28 dpi agroinoculated with wild‐type CaLCuV. (h) Col‐0 inoculated with wild‐type CaLCuV at 28 dpi.

A total of 166 accessions were bombarded with CaLCuV:CH‐42 to assess both symptoms and silencing. Each accession was placed into one of four classes based on the extent of silencing and symptom severity (Figure 1; Table S1): Class A, most of the accessions with significant symptoms and silencing, and those with a weak VIGS response; Class B, accessions with low symptoms and high silencing – that would be a suitable host for VIGS; Class C, accessions with very low silencing but severe symptoms – interesting because of a lack of VIGS; and Class D, accessions with very low symptoms and very low silencing – candidates for resistance. Figure 1 shows examples of accessions from each of the classes, and individual photos of the phenotypic response of each accession, grouped by experiment, are shown in Figure S1. Col‐0 consistently received high scores for both symptoms and the extent of silencing in each of the experiments (Table S1; Figure S1).

Class A had 144 members and included all accessions not placed in the other classes. These accessions had moderate to severe symptoms (138 accessions) or, if mild symptoms, limited silencing (six accessions). Only 20 of these lines had severe symptoms, but strikingly none of them showed an extensive VIGS response (Table S1; Figure S1). Class C had three accessions that also showed severe symptoms, but VIGS in these lines was greatly reduced or in some cases absent.

Class B contained 10 accessions with extensive silencing but minimal symptoms. Several accessions stood out for their robust silencing response and attenuated symptoms throughout development. The best VIGS responses were found in Kil‐0 (Figure 1b), Le‐0, Ka‐0 and Gu‐1, followed by Ra‐0 and Sf‐2 (Figure S1). Some accessions, in Class A because of their phenotype at ~25 dpi, showed attenuated symptoms at later time points, similar to recovery in wild‐type virus infections, and are marked with an asterisk in Table S1. Figure S2 shows three representative accessions that had stunted inflorescences at 26 dpi. At 45–60 days, symptoms attenuated, leaf sizes increased and inflorescences developed normally and the plants showed increased silencing.

Class D had six members that showed resistance to the VIGS vector but would need to be retested with wild‐type CaLCuV to assess resistance. The bottom row of Figure 1 shows two exceptional examples of Class D resistance. In the first example, plants such as Li‐2:1 showed an early VIGS response (Figure 1e) but then appeared to recover to become symptomless (Figure 1f). All five of the Li‐2:1 plants showed the same pattern (Figure S1). Another accession, PNA‐17, also showed a transient VIGS response except that silenced areas were light green rather than yellow (Figure S1). These were the only accessions that lost the VIGS phenotype.

The second example, the Pla‐1 accession (ecotypeID 7301) from Playa de Aro in Spain, never showed symptoms or silencing. Moreover, no signs of infection were observed when Pla‐1 plants were agroinoculated with wild‐type CaLCuV, which contains the viral coat protein gene and produces much more severe symptoms than CaLCuVA:CH‐42 (Figure 1g).

### Lack of viral DNA accumulation in Pla‐1

**Figure 2:**
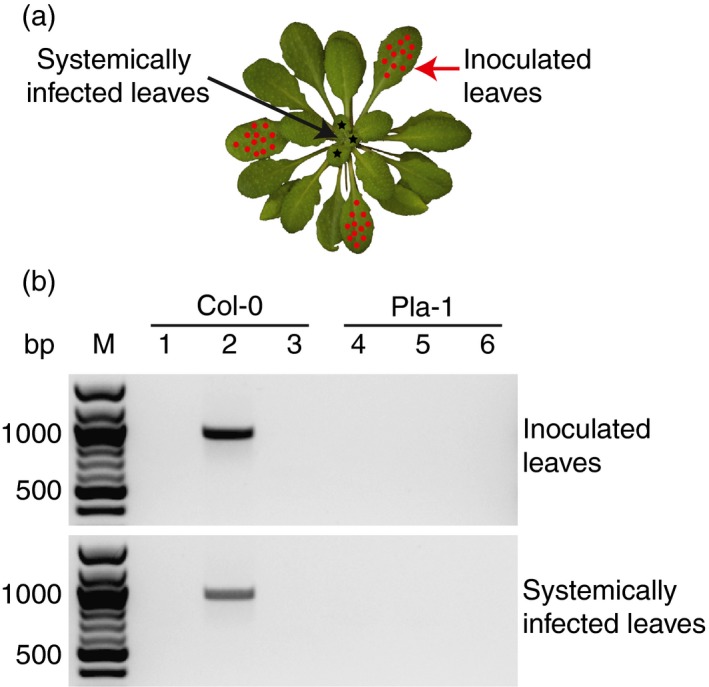
Pla‐1 is immune to wild‐type CaLCuV. (a) An Arabidopsis plant illustrating the three leaves that were inoculated separately (red dots) and leaves in new growth that were tested for systemic infection (black stars). Photo modified from Charles Andrès (licensed under CC BY 2.0). (b) Polymerase chain reaction (PCR) detection of viral DNA in inoculated (upper panel) and systemically infected leaves (lower panel) of Col‐0 and Pla‐1. Lanes 1 and 4 show mock‐inoculated plants, lanes 2 and 5 show plants inoculated with wild‐type CaLCuV, and lanes 3 and 6 show plants inoculated with the CaLCuV replication‐deficient mutant. Lane M shows DNA size markers. The expected PCR product is 936 bp.

Successful infection by geminiviruses requires viral DNA replication and gene expression as well as nuclear, cellular and long‐distance movement (Morra and Petty, 2000; Trejo‐Saavedra *et al*., 2009). To identify the stage where infection was blocked, we inoculated individual leaves of Pla‐1 and Col‐0 (Figure 2a). A single leaf was inoculated by microprojectile bombardment with wild‐type CaLCuV, and the process repeated until three mature leaves from the same plant were inoculated. Symptoms appeared in young, developing leaves of Col‐0 at 13 dpi. Leaf curling, stunted development and mild chlorosis were present at 17 dpi and pronounced chlorosis at 22 dpi. In contrast, Pla‐1 did not develop symptoms.

We tested for viral DNA accumulation using polymerase chain reaction (PCR) primers that were divergent in pCPCbLCVA.003, a plasmid containing partial tandem copies of wild‐type A DNA with duplicated 5′ intergenic regions. The primers were expected to produce a 936‐bp product from replicated viral DNA in infected tissues and a ~4.6‐kb product from the input plasmid. A replication‐deficient mutant containing a frameshift mutation in *AL1*, which is essential for viral DNA replication (Elmer *et al*., 1988; Sunter *et al*., 1990), was tested to distinguish between homologous recombination between the duplicated 5′ intergenic regions of input DNA and low levels of authentic viral DNA replication. The *AL1* mutant did not replicate (Figure S3) in protoplast assays (Methods S1).

Viral DNA was detected in inoculated leaves of Col‐0 at 9 dpi and in systemically infected leaves at 13 dpi. Figure 2b shows viral DNA accumulation at 22 dpi. No viral DNA was detected in Col‐0 inoculated with the *AL1* mutant, suggesting that viral DNA replication *in planta* was required for detection by our PCR primers. In Pla‐1, none of the DNAs, including wild‐type, was detected (Figure 2b). These results show that at least part of the Pla‐1 resistance impacts infection at or before viral DNA is replicated, and establish that Pla‐1 is immune to CaLCuV.

### Pla‐1 is also immune to a *Curtovirus*

**Figure 3:**
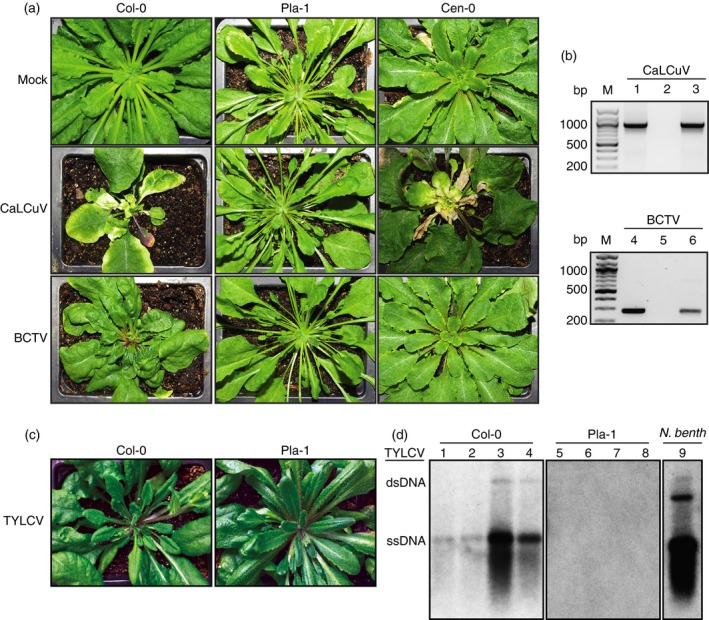
Pla‐1 is resistant to agroinoculation with CaLCuV, BCTV and TYLCV. (a) Symptom development at 25 dpi in Col‐0, Pla‐1 and Cen‐0 plants agroinoculated with CaLCuV or BCTV. (b) Polymerase chain reaction (PCR) detection of viral DNA in Col‐0 (lanes 1, 4), Pla‐1 (lanes 2, 5) or Cen‐0 (lanes 3, 6) plants inoculated with CaLCuV (lanes 1–3) or BCTV (lanes 4–6). Lane M shows DNA size markers. The expected PCR product size for CaLCuV is 936 bp and for BCTV is 283 bp. (c) Symptom development at 35 dpi in Col‐0 and Pla‐1 plants agroinoculated with TYLCV. (d) DNA blot hybridization of total DNA from TYLCV inoculated Col‐0 (lanes 1–4), Pla‐1 (lanes 5–8) and the positive control *Nicotiana benthamiana* (lane 9) at 35 dpi. The blot was probed with P^32^‐labeled TYLCV DNA and imaged on film. Lanes 1–4 contain DNA from Col‐0 plants showing the mildest (lanes 1 and 2) and most severe (lanes 3 and 4) symptoms out of eight inoculated plants. dsDNA, double‐stranded DNA. ssDNA, single‐stranded DNA.

To test whether Pla‐1 resistance extends to other geminiviruses, we challenged Pla‐1 plants with *Beet curly top virus* (BCTV), a member of the *Curtovirus* genus. Because BCTV is phloem‐limited and not efficiently inoculated by bombardment (Briddon *et al*., 1989; Stenger *et al*., 1990), we agroinoculated both CaLCuV and BCTV. Pla‐1 inoculated with BCTV (Figure 3a) did not show symptoms. It has been previously reported that another accession, Cen‐0, was resistant to BCTV (Park *et al*., 2002). We confirmed that Cen‐0 had no symptoms from BCTV but it was susceptible to CaLCuV and displayed similar symptoms as Col‐0 – severe chlorosis and leaf deformation (Figure 3a). Symptoms in BCTV‐infected Col‐0 included leaf curling and deformation plus anthocyanin accumulation, but chlorosis was not apparent.

To test for immunity to BCTV, we used PCR (Figure 3b). Col‐0 inoculated with CaLCuV and BCTV showed bands of the predicted sizes indicating that viral DNA was present in systemically infected leaves. However, neither CaLCuV nor BCTV produced detectable viral DNA in Pla‐1. CaLCuV DNA accumulated in Cen‐0 at levels comparable to Col‐0, consistent with the symptoms. Despite the lack of symptoms in Cen‐0, BCTV DNA was detected in systemic growth, confirming previous results that Cen‐0 is tolerant to BCTV (Park *et al*., 2002). These results demonstrate that Pla‐1 has broad‐based immunity to geminiviruses and that Cen‐0 tolerance is distinct from Pla‐1 immunity.

### Pla‐1 shows resistance to TYLCV infection

Recently, Col‐0 was shown to be susceptible to both the severe, Israel strain (IL), and the mild strains of TYLCV (Cañizares *et al*., 2014). Because of the severe crop losses caused by TYLCV globally (Diaz‐Pendon *et al*., 2010), we decided to test for susceptibility in Pla‐1. We agroinoculated eight Col‐0 and seven Pla‐1 plants with a variant of the Israel strain that came from the Dominican Republic (TYLCV‐IL[DO]). Eight *Nicotiana benthamiana* plants, a known host for TYLCV, were also agroinoculated as a positive control for infection. TYLCV symptoms were clearly present in *N. benthamiana* by 10 dpi, but were not apparent in Col‐0 or Pla‐1. Mild symptoms appeared in Col‐0 at 21 dpi, while Pla‐1 did not show symptoms, even at 35 dpi when the plants were used for DNA extraction (Figure 3c). DNA:DNA blot hybridization of genomic DNA from the infected plants with a TYLCV probe showed that viral DNA was present in systemically infected leaves of both *N. benthamiana* and each of the eight inoculated Col‐0 plants. In contrast, there was no hybridization with DNA from any of the seven inoculated Pla‐1 plants (Figure 3d). Together with the lack of symptoms, we conclude that Pla‐1 is resistant to TYLCV.

### Pla‐1 is susceptible to RNA viruses

**Figure 4:**
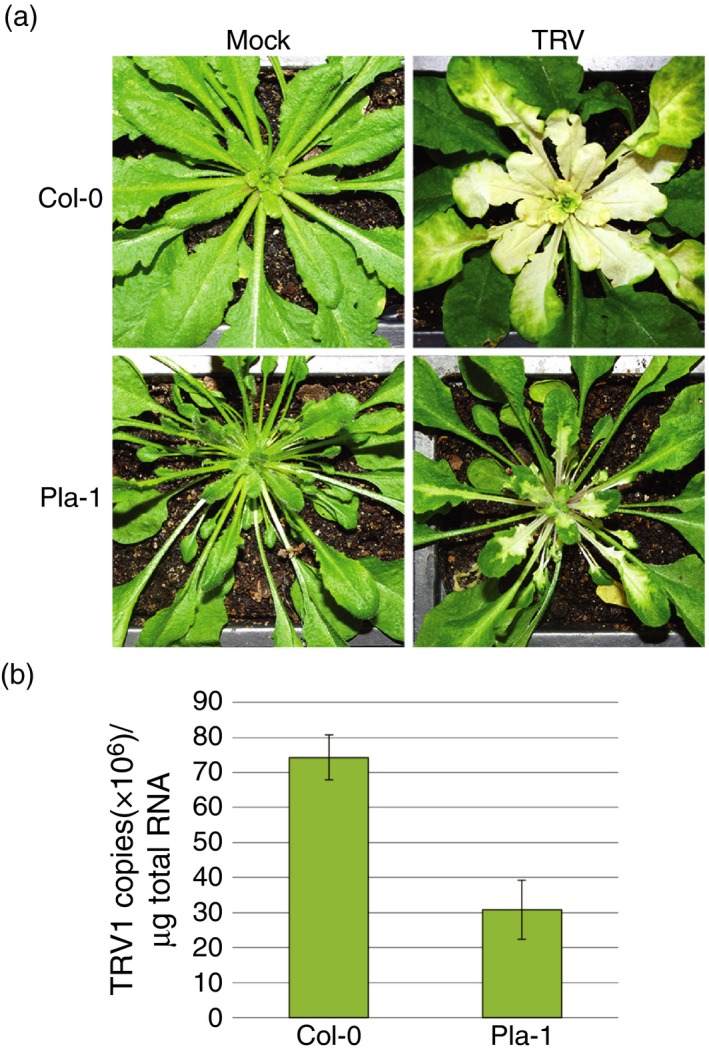
Pla‐1 shows reduced virus‐induced gene silencing (VIGS) and susceptibility to TRV:*AtPDS*. (a) Response of Col‐0 and Pla‐1 to TRV:*AtPDS* inoculation at 21 dpi. (b) Histogram of TRV1 copy number per μg of total RNA for Col‐0 and Pla‐1 TRV:*AtPDS*‐inoculated plants. Error bars show standard error.

To determine if Pla‐1 immunity was specific to DNA viruses, we tested Pla‐1 for infection with the RNA tobravirus *Tobacco rattle virus* (TRV). Because TRV infection is asymptomatic (Burch‐Smith *et al*., 2006), and we were also interested in the VIGS response of Pla‐1, we used TRV carrying a visible marker for silencing, a fragment of *Phytoene Desaturase* (*AtPDS*; At4G14210; Figure 4). PDS is needed for carotenoid biosynthesis, which protects chlorophyll from photobleaching. Although extensive *AtPDS* silencing was observed in Col‐0, the extent of VIGS was reduced in Pla‐1, and only five out of six plants showed silencing (Figure 4a). A second experiment showed that new growth in TRV:*AtPDS*‐inoculated Pla‐1 was green at later time points while the equivalent leaves in Col‐0 plants remained white (Figure S4).

To determine whether the reduced TRV:*AtPDS* VIGS in Pla‐1 was due to a defective silencing response or reduced TRV susceptibility, viral RNA accumulation was analyzed using TaqMan^®^. pTRV1 contains the viral RDR but lacks the *AtPDS* silencing fragment. The copy number of TRV1, which was normalized to total RNA (μg), was significantly higher in Col‐0 plants (69 × 10^6^) compared with Pla‐1 plants (26 × 10^6^; *P* < 0.05; Figure 4b). Therefore, Pla‐1 shows some resistance to the TRV:*AtPDS* VIGS vector in comparison to Col‐0. Because the reduced silencing could be a direct effect of reduced TRV:*AtPDS* levels, we were not able to conclude whether the silencing response was altered in Pla‐1.

Because Pla‐1 showed reduced susceptibility to TRV:*AtPDS*, we challenged Pla‐1 plants with another RNA virus, the potyvirus *Turnip mosaic virus* (TuMV; Methods S2). All eight TuMV‐inoculated Pla‐1 plants showed severe symptoms at 18 dpi, indicating susceptibility (Figure S5).

### Immunity maps to chromosome 1

To determine if Pla‐1 immunity corresponded to previously identified resistance genes, we initiated QTL mapping with progeny from a Pla‐1 × Col‐0 cross. In the first set of experiments, 83 F_2:3_ families were agroinoculated with wild‐type CaLCuV and scored for symptoms at 21 dpi in three replicates (Methods S3). Symptoms were scored on a scale of 1–5 (Figure S6), with 1 being no symptoms and 5 being severe chlorosis, stunting and growth arrest. A total of 20 simple sequence length polymorphism (SSLP) markers polymorphic for Pla‐1 and Col‐0 (Table S2; Figure S7c) were used to construct a QTL map using R/qtl (Broman and Sen, 2009). Plots for the three replicates showed a major peak on chromosome 1 near nga280 (Figure S7d) with logarithm of odds (LOD) scores of about 5.5. A LOD threshold of 3.5 was calculated from 1000 permutations at 0.05 confidence, indicating that the peaks were significant. The consistency of results from different members of the F_2:3_ families in three replicates showed that resistance was heritable and suggested that fine‐mapping should concentrate on chromosome 1.

To identify single nucleotide polymorphisms (SNPs) for chromosome 1, the Pla‐1 genome was sequenced to a depth of 3 ×. Although coverage was low, many SNPs were supported by at least 5 reads. A total of 77 SNPs near nga280 were chosen from Pla‐1 sequence and supplemented with 19 previously identified SNPs (Platt *et al*., 2010) to provide complete coverage of the genome. Illumina's Golden Gate technology was used to identify and call SNPs, and oligonucleotides for the 96 putative SNPs passed Illumina's proprietary criteria for inclusion in the assay. Unfortunately, more than 50% of the SNPs failed to produce useful data, largely due to incomplete data for the Pla‐1 genome. Sequences and positions of the 42 successful SNPs are provided in Table S3.

**Figure 5:**
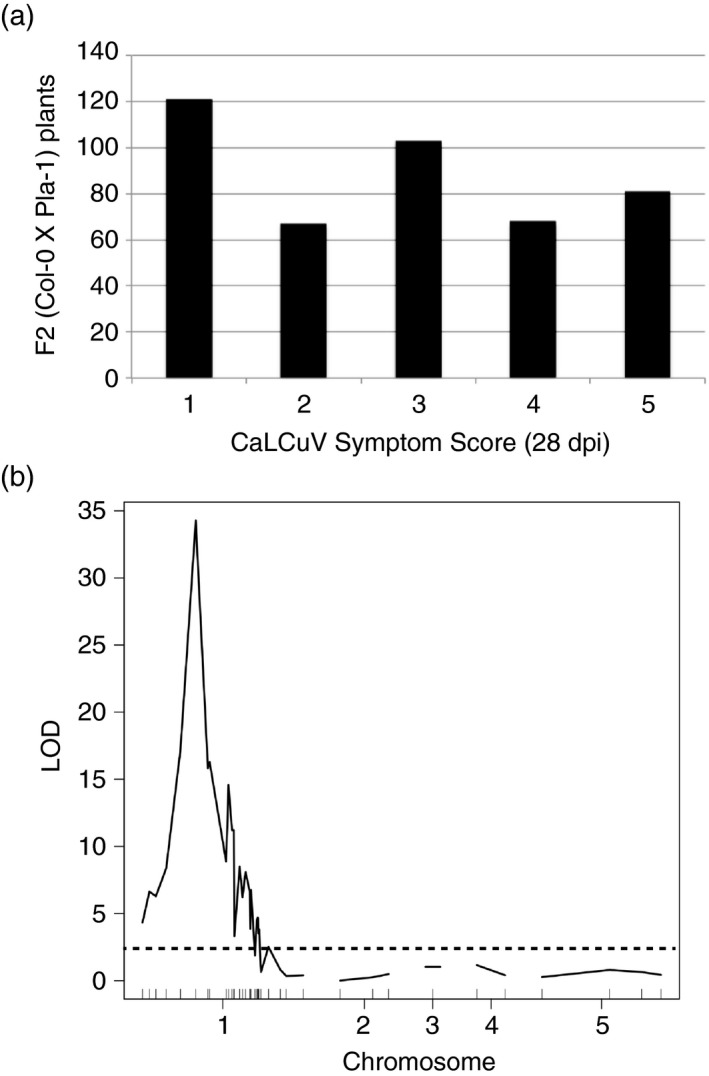
Quantitative trait locus (QTL) associated with CaLCuV symptoms are located on chromosome 1 of Pla‐1. (a) 28‐dpi symptom responses of the 440 F_2_ (Pla‐1 × Col‐0) plants used for mapping to CaLCuV. (b) QTL map of symptom responses in the CaLCuV‐inoculated F_2_ population. Positions of the single nucleotide polymorphism (SNP) markers along the chromosomes are shown as vertical black lines on the *x*‐axis. The black dashed line marks the logarithm of odds (LOD) significance threshold (*P* < 0.05).

A total of 440 F_2_ progeny were agroinoculated with CaLCuV, scored for symptoms at 28 dpi and used for the second QTL map. Of the 440 plants, 121 plants (27.5%) showed no symptoms (symptom score 1; Figure 5a). The ratio of resistant (121) to susceptible (319) plants was similar to the expected ratio (1:3) for recessive resistance with a chi square value of 1.47 (*P* = 0.23 that expected and observed were from different populations). These results were similar to the F_2:3_ families (Figure S7b), and indicate that immunity is recessive.

The positions of the 42 SNPs used to genotype 440 plants are shown in Figure 5b. R/qtl was used to create a QTL map using extended Haley–Knott regression (Feenstra *et al*., 2006). The results show a major QTL, designated *gip‐1* for geminivirus immunity, Pla‐1, at 42.6 centiMorgan (cM) in chromosome 1 with a LOD score of 34 (Figure 5b). Following 1000 permutations, the LOD score for 0.05 significance was 2.85. Similar results were obtained using standard interval mapping (Lander and Botstein, 1989) and the original Haley–Knott regression method. These results located a major QTL on the left side of the centromere that contrasts with results from F_2:3_ families, which showed a peak to the right of the centromere. This can be explained by the paucity of SSLP markers in the center of chromosome 1, which likely skewed results from the F_2:3_ families. Both analyses found a major peak on chromosome 1. In addition, both studies showed that QTLs on other chromosomes do not have a strong impact on the production of CaLCuV symptoms. From these results, we conclude that Pla‐1 immunity is distinct from the RDR‐like *Ty‐1/Ty‐3* or the Pelota‐like *ty‐5* genes previously identified in tomato, which are located in chromosomes 2 and 4, respectively (Verlaan *et al*., 2013; Lapidot *et al*., 2015). Therefore, the gene(s) responsible for Pla‐1 immunity are likely to be novel. Candidate genes near *gip‐1*, whose sequences are altered compared with the geminivirus‐susceptible accession Col‐0 and the closely related susceptible accession Pla‐0, are listed in Table S4. This list includes 582 SNPs unique to Pla‐1 located in the open reading frames of 161 genes (Methods S4).

## Discussion

This work began as an effort to identify a suitable host for geminivirus‐mediated VIGS in Arabidopsis, but ultimately focused on resistance. After screening 190 Arabidopsis accessions, we identified Pla‐1 as the only accession immune to CaLCuV, a whitefly‐transmitted bipartite member of Squash leaf clade in the Begomovirus genus of Geminiviridae. Pla‐1 was also resistant to BCTV, a leafhopper‐transmitted monopartite geminivirus in the *Curtovirus* genus, and to the agronomically important TYLCV, a whitefly‐transmitted monopartite member of the Old World branch of the Begomovirus genus, thereby establishing the broad‐based nature of the immunity.

Pla‐1 was inoculated with wild‐type CaLCuV by microprojectile bombardment into three leaves of the same plant (Figure 2) or by agroinoculation (Figure 3). In both cases, no viral DNA was recovered in inoculated or systemically infected leaves. In addition, no viral DNA was detected when Pla‐1 was inoculated with BCTV. Furthermore, TYLCV could not be detected in any of the inoculated Pla‐1 plants. This kind of broad‐based immunity is desirable in plant breeding programs because the lack of viral DNA accumulation reduces the chances of viral variants with the potential to break resistance.

Recessive resistance, especially when it is to more than one genus of virus, suggests that a host protein(s) essential for infection is altered or unavailable to the virus (Pagny *et al*., 2012; Ouibrahim *et al*., 2014; Lapidot *et al*., 2015). DNA viruses exploit the host's capacity for protein and nucleic acid synthesis, nuclear trafficking, and cell‐to‐cell and long‐distance movement (Ascencio‐Ibanez *et al*., 2008; Hanley‐Bowdoin *et al*., 2013). Although we could not detect viral DNA in inoculated leaves, our assay may not have been sensitive enough to detect very low‐level DNA replication. We included an AL1 frameshift mutant because episomes were detected when a similar construct for *Tomato golden mosaic virus* was transfected into *Escherichia coli* (Lopez‐Ochoa *et al*., 2006). These episomes likely formed by recombination across the duplicated 5′ intergenic regions of input plasmid, but it is not known whether episomes can be formed simply by homologous recombination in plant cells. Therefore, we do not know the lower limits of detection in our assay and can not rule out the occurrence of very low levels of viral DNA replication. However, when wild‐type CaLCuV A is bombarded into mature leaves in the absence of B DNA, which is essential for movement, viral DNA is easily detected. Therefore, at least part of the Pla‐1 immunity must involve host processes that target early events in the viral life cycle.

The VIGS screen of Arabidopsis accessions resulted in a variety of responses, but two correlations were found – lines that showed reduced symptoms over time (recovery) also showed increased VIGS, and lines that showed the most severe symptoms lacked extensive silencing (Table S1). Figure S2 shows the extensive VIGS associated with recovery in some of the accessions at later time points. One explanation for the increased VIGS response is that transcriptional gene silencing (TGS), which methylates viral DNA, stops transcription of viral genes that inhibit silencing. Geminiviruses encode multifunctional anti‐silencing proteins that target TGS as well as PTGS (Wang *et al*., 2005; Glick *et al*., 2008; Rodriguez‐Negrete *et al*., 2009). Recovery from geminivirus infection has been correlated with increased methylation of viral DNA (Raja *et al*., 2008), but viral DNA is not entirely eliminated in new growth and not all DNAs are methylated (Paprotka *et al*., 2011). If only a few DNAs are transcriptionally active, anti‐silencing protein levels may be reduced compared with early stages of infection. Any transcription of the *CH‐42* insert could be amplified by RDR6 and cause extensive silencing in new growth.

Viral anti‐silencing activity may be especially strong against both TGS and PTGS in other accessions, such as those in Class C that show severe symptoms and very limited PTGS. Previous studies have identified RDR6, SGS3, DCL4 and Hen1 as necessary for VIGS from CaLCuV in Arabidopsis (Blevins *et al*., 2006). Plants mutant for *RDR6* and *SGS3*, which encodes a known target of geminivirus anti‐silencing proteins (Glick *et al*., 2008), also show more severe symptoms and reduced silencing (Muangsan *et al*., 2004). The strong correlation between severe symptoms and reduced VIGS suggests that the relative strength of the host gene silencing defense response determines symptom severity in this virus host pathosystem.

Several accessions showed minimal symptoms and extensive silencing. Accessions in Class B, especially Gu‐1, Kil‐0, Le‐0, Sf‐2, Mz‐0 and Ra‐0, could be suitable hosts for VIGS as a functional genomics tool, depending on the goals of the research (Flores *et al*., 2015). We used relatively cool (20–22°C) growth conditions. Because the extent of VIGS increases with temperature (Chellappan *et al*., 2005; Wang *et al*., 2006; Tuttle *et al*., 2008), increased silencing might be obtained for some of these accessions if grown at higher temperatures.

The response of plants in Class D, which showed reduced VIGS as well as reduced symptoms, could reflect mutations in genes for essential host factors, such as *Pelo*. In *ty‐5*, which has an alternative *Pelo* allele, TYLCV viral DNA levels are greatly reduced and infection is asymptomatic (Lapidot *et al*., 2015). However, the lines in Class D need to be screened with wild‐type CaLCuV before they are considered to be resistant.

Only one out of 190 accessions (Pla‐1) showed a complete lack of symptoms and silencing when inoculated with the VIGS vector, suggesting that VIGS is a sensitive method for distinguishing between resistance and immunity. Arabidopsis is susceptible to a variety of viruses and several screens for resistant accessions have been reported (Leisner and Howell, 1992; Martín Martín *et al*., 1997; Park *et al*., 2002; Rajakaruna and Khandekar, 2007; Ouibrahim *et al*., 2014). However, the only report of virus immunity in Arabidopsis described a Col‐0 mutant already resistant to tobamoviruses that was subjected to further mutagenesis (Yamanaka *et al*., 2002). Further testing will be needed to determine whether one or more genes comprise the Pla‐1 immunity to CaLCuV and whether this accession is unique in showing broad‐based immunity to geminiviruses.

The only other study to test the response of Arabidopsis accessions to a VIGS vector, which used the same TRV:*AtPDS* as in Figure 4, found very little variation in symptoms or the extent of silencing (Wang *et al*., 2006). In contrast, we found wide variation in both symptoms and the extent of silencing. We also found a reduction in the extent of VIGS from TRV:*AtPDS* in Pla‐1 compared with Col‐0, but this may have been due to reduced levels of the TRV vector. Although resistant to CaLCuV, BCTV, TYLCV and perhaps to some extent TRV, Pla‐1 has been shown to be very susceptible to *Cauliflower mosaic virus*, a DNA virus that replicates through RNA intermediates (Leisner and Howell, 1992), the potyvirus TuMV (Figure S5), and the comovirus *Turnip ringspot virus* (Khandekar *et al*., 2007).

A recessive susceptibility locus, *sha3*, that impacts long‐distance movement of the *Potyvirus Plum Pox Virus* (PPV) was mapped to a 20‐kb region on chromosome 3 by combining GWAS of 147 Arabidopsis accessions and traditional QTL mapping (Pagny *et al*., 2012). Six different accessions showed resistance to PPV and were allelic for *sha3*. Our initial attempts at using GWAS have not yielded clear results for *gip‐1*, perhaps because the wild‐type CaLCuV virus was not used or, more likely, because immunity is polygenic and one or more alleles are not prevalent among other accessions.

Accessions related to Pla‐1, except for Pla‐0, showed a reduction in either symptoms or silencing (Figure S1). Only Pla‐0 has been sequenced (Consortium, T.G, 2016), and its genome will be useful for eliminating candidate genes. Pla‐2 and Pla‐3 both showed mild symptoms. Although Pla‐4 had strong symptoms, it was placed in Class C due to the lack of a significant VIGS response (Table S1). These VIGS responses demonstrate significantly more viral DNA replication and movement than found in Pla‐1. Efforts are currently underway to transfer *gip‐1* to Col‐0 to determine if immunity can be retained in a different background. These efforts are complicated by the longer flowering time for Pla‐1 and the need to vernalize F_1_ progeny to induce flowering.

Resistance genes in tomato have been identified for TYLCV, which is in the same genus as CaLCuV (Begomovirus), but has a single‐component genome and is limited to the phloem during infection. The *Ty‐1*/*Ty‐3* alleles have alterations in an *RDR* that shows high sequence similarity to Arabidopsis *RDR3*,* RDR4* and *RDR5* (Verlaan *et al*., 2013), all of which are located on chromosome 2. The recessive mutation in *Pelo*, at the *ty‐5* locus, confers strong resistance to TYLCV, probably by inhibiting or slowing down ribosome recycling and reducing protein synthesis in the infected cells (Lapidot *et al*., 2015). The corresponding gene in Arabidopsis is located in chromosome 4 and is distinct from the Pla‐1 resistance locus on chromosome 1. The *Ty‐1* and *Ty‐3* alleles behave differently: *Ty‐1* is dominant and is specific for TYLCV; while *Ty‐3* is semi‐dominant and also confers partial resistance to the bipartite *Tomato mottle virus,* and the combination of alleles provides stronger resistance than either allele alone (Ji *et al*., 2007). The *ty‐5* allele is recessive and provides strong protection against TYLCV but may be associated with reduced growth in the absence of infection (Lapidot *et al*., 2015). Nevertheless, recessive resistance that involves alterations in essential host factors is hard for the virus to overcome. Identification of the genetic basis for the immunity found in Pla‐1, which is also recessive, could provide information about another virus–host interaction that can be targeted for resistance. An advantage of using natural variation is that the genes conferring resistance have been selected for fitness, which is important because these viruses target essential host processes. The possibility of using CRISPR/Cas technologies to precisely modify the corresponding genes in crop plants without the need for traditional plant transformation (Puchta, 2016) may speed the deployment of these genes where they are most needed. Candidate genes for conferring geminivirus immunity in the *gip‐1* locus are listed in Table S4. The list includes genes that encode proteins involved in pathogen and stress responses, transcription, hormonal regulation and development. All of these pathways have been implicated in geminivirus infection (Hanley‐Bowdoin *et al*., 2013) and their disruption has the potential to interfere with the geminivirus infection process.

Tagging CaLCuV with a marker for VIGS was useful in uncovering dynamic aspects of geminivirus–host interactions that would have been difficult to track in a wild‐type virus infection. It was especially useful in identifying CaLCuV immunity in Pla‐1. A single peak was identified by QTL mapping of CaLCuV‐infected plants, but it still may consist of more than one gene. Still to be determined is whether the CaLCuV immunity in Pla‐1 comprises a unique combination of genes that are also present in other accessions or if it includes a rare allele. It also needs to be established whether *gip‐1* participates in resistance to BCTV and/or TYLCV. Nevertheless, the high LOD score of *gip‐1*, the block of viral DNA accumulation from two distinct geminiviruses in Pla‐1 and the resistance shown against TYLCV all suggest that further analyses will be valuable. Efforts are currently underway to identify the molecular basis of Pla‐1 immunity.

## Experimental procedures

### Plant growth

Seeds were stratified at 4°C for 3 days on moist autoclaved Metro‐Mix 360 soil. For the VIGS screen, plants were grown at 22/20°C during an 8‐h light/16‐h dark photoperiod. For TYLCV agroinoculation, plants were grown in continuous light at 22°C. All other experiments used plants grown at 20°C under an 8‐h light/16‐h dark photoperiod with 50% humidity at a light intensity of 140 μmol m^−2^ sec^−1^.

### Plasmid construction

All plasmids for geminivirus inoculation carried duplicated 5′ intergenic regions for replicational release in plant cells (Elmer *et al*., 1988). Plasmids carrying the wild‐type CaLCuV A DNA (pCPCbLCVA.003), the CaLCuV A DNA VIGS vector (CaLCuVA:CH‐42 with a 362‐bp fragment of *CH‐42* in antisense orientation in place of the coat protein gene, pMTCaLCuVA.008), the CaLCuV A DNA LUC vector (CaLCuVA:LUC with a 623‐bp fragment of *LUC*, pNMCaLCuVA.LUC) and wild‐type CaLCuV B DNA (pCPCbLCVB.002) have been described (Turnage *et al*., 2002; Muangsan *et al*., 2004). The replication‐deficient CaLCuV A mutant (pCaLCuVA:FSAL1mut) carrying a frameshift mutation in *AL1* was created by digesting pCPCbLCVA.003 with *Nco*I, repairing the cleaved ends and religating it. CaLCuV‐containing Agrobacterium plasmids have been described (Egelkrout *et al*., 2002). The BCTV‐Logan (Stenger *et al*., 1991) plasmid was provided by D.M. Bisaro of The Ohio State University. TRV plasmids were obtained from the Arabidopsis Biological Resource Center (ABRC, Ohio State University). The plasmid carrying TYLCV‐IL[DO] (GenBank accession number AF024715) has been described (Reyes *et al*., 2013).

### CaLCuV VIGS of Arabidopsis accessions

Of the 190 accessions screened, 173 came from ABRC and were bulked at Paradigm Genetics and 17 were from the collection of 96 natural accessions (Nordborg *et al*., 2005) obtained from ABRC as CS22660. Seedlings at the seven‐eight leaf stage were co‐bombarded with equal amounts of CaLCuVA:CH‐42 or CaLCuVA:LUC and CaLCuV B, as previously described (Turnage *et al*., 2002). Twenty seedlings per accession were bombarded and 16 accessions were screened simultaneously along with Col‐0 controls. Four people independently evaluated symptoms, and silencing and consensus scores (Table S1) were reached by group discussion.

### Microprojectile bombardment of individual leaves with CaLCuV

Equal amounts (2.5 μg) of the wild‐type or replication‐deficient CaLCuV A and CaLCuV B DNAs were precipitated onto 1‐μm gold microprojectiles (Santos *et al*., 2008) and co‐inoculated three times into three adjacent mature rosette leaves using a DNA microsprayer (Venganza) at 30 psi. The experiment was repeated twice and included CaLCuV B‐inoculated controls.

### CaLCuV, BCTV and TYLCV agroinoculation and detection

Five‐week‐old seedlings were inoculated with an equal mixture of Agrobacterium carrying CaLCuV A and B plasmids, the BCTV or the TYLCV containing plasmid (Ascencio‐Ibanez *et al*., 2008). Agrobacterium with an empty vector served as control. Agrobacterium cultures were grown overnight at 30°C until saturation. Plants were inoculated by pricking the area surrounding the shoot apex 10 times and depositing a drop of the culture using a 1‐mL syringe with a 27.5‐gage needle. Plants were then covered for 24 h. Leaves 6 and 7 (with leaf 1 being the youngest) were pooled at 25 or 35 dpi for genomic DNA extraction using the DNeasy Plant Mini Kit (Qiagen) or a Plant/Fungi DNA isolation kit (Norgen). CaLCuV DNA was detected by PCR using divergent primers CaLCuVAdivPCR‐For 5′‐ CTCTAGGAACATCTGGGCTTCTA and CaLCuVAdivPCR‐Rev 5′‐ CCTTATAATTGCGAGACGCTCT. BCTV DNA was detected using primers BCTV15‐for 5′‐CGTTACTGTGACGAAGCATTTG and BCTV15‐rev 5′‐CTCCTTCCCTCCATATCCAGTA. All assays were run in triplicate and included no DNA and target DNA controls. TYLCV was detected by DNA blot hybridization using 100 ng of genomic DNA digested with *Sac*I. Blotted membranes were hybridized to an [α‐^32^P]‐dATP labeled 1619‐bp TYLCV *Cla‐*1 fragment and exposed to CL‐X Posure film.

### TRV VIGS

Arabidopsis plants at the 12–14 leaf stage were used for TRV VIGS. pTRV1 and pTRV2‐*AtPDS* were introduced into *Agrobacterium tumefaciens* strain GV3101::pMP90 by electroporation and equal amounts were mixed together before infiltration. Agroinfiltration of six plants per accession (Col‐0 and Pla‐1) was performed as described previously (Burch‐Smith *et al*., 2006).

### TRV quantification

Three PDS‐silenced leaves were pooled per plant. Total RNA was isolated using an RNeasy Plant Kit (Qiagen, Crawley, UK). First‐strand cDNA synthesis was performed using a SuperScript III kit (Invitrogen, Paisley, UK) and the Tobravirus‐specific primer 305 5′‐GGGCGTAATAACGCTTACG.

Primers and probes derived from the 3′ ORF in TRV RNA1 were used as described (Holeva *et al*., 2006). The 5′ reporter dye was FAM and the 3′ dye was TAMRA (Applied Biosystems). Real‐time PCR was performed using the Mx3000P qPCR System (Stratagene). Each assay was performed in triplicate and included either no cDNA template or pTRV1 controls. TRV1 in 10‐fold dilutions (10 ng to 100 fg) were run in triplicate as standards for quantification. Crossing threshold (*C*t) values were calculated by MxPro QPCR Software (Agilent). TRV1 copy number was calculated using the following formula: (g of TRV1 DNA/(size of TRV1 DNA in bp)/molecular weight of 1 bp) * Avogadro's constant.

### Genotyping and QTL mapping

To identify markers, the Pla‐1 genome was sequenced using Illumina Sequencing by Synthesis technology at NCSU's Kannapolis campus. DNA was isolated from Pla‐1 ecotypeID 7301 using a Qiagen DNeasy kit. About 15 million reads with an average length of 33 bp were aligned to the TAIR 9 version of Col‐0 using Bowtie2‐2.1.0 (Langmead *et al*., 2009). SNPs were called using SAMTools (Li *et al*., 2009) and 96 SNPs were used by Illumina (San Diego, CA, USA) to design oligonucleotides for the GoldenGate Genotyping assay with VeraCode Technology.

A total of 440 5‐week‐old F_2_ plants, plus Col‐0 and Pla‐1 controls, were scored for wild‐type CaLCuV infection (Figure S6). Three young leaves from each plant were used for DNA extraction (Stepanova *et al*., 2011). DNA was quantified using PicoGreen^®^ (Life Technologies), and 15 μL at 50–100 ng μL^−1^ was sent to the Genomics Core at Case Western Reserve School of Medicine for processing and calling SNPs.

Quantitative trait locus analyses was performed using R/qtl [R version 3.2.2 (2015‐08‐14); Broman and Sen, 2009] with extended Haley–Knott regression. LOD thresholds were determined by performing 1000 permutations to estimate the 0.05 significance level.

## Conflict of Interest

The authors declare no conflict of interest.

## Supporting information


**Figure S1.** Thumbnail images of *CH‐42* VIGS in different Arabidopsis accessions.

**Figure S2.** Examples of accessions with attenuated symptoms and increased silencing over time.

**Figure S3.** CaLCuV *AL1* frameshift mutation abolishes viral DNA replication in *Nicotiana tabacum* (NT1) protoplasts.

**Figure S4.** New growth in Pla‐1 lacks TRV:*AtPDS* VIGS at later time points compared with Col‐0.

**Figure S5.** Pla‐1 is susceptible to TuMV.

**Figure S6.** CaLCuV symptom score key.

**Figure S7.** QTL maps from F_2:3_ families.

**Table S1.** Response of 190 Arabidopsis accessions to inoculation with the CaLCuVA:CH‐42 VIGS vector or to CaLCuVA:LUC

**Table S2.** SSLP markers for Pla‐1 and Col‐0

**Table S3.** SNPs for Pla‐1 and Col‐0

**Table S4.** Candidate Genes for *geminivirus immunity Pla‐1‐1* (*gip‐1*)

**Methods S1.** CaLCuV A DNA replication assay in *Nicotiana tabacum* (NT1) protoplasts.
**Methods S2.** TuMV inoculation.
**Methods S3.** QTL mapping using F_2:3_ families.
**Methods S4.** Generation of the *geminivirus immunity* candidate gene list.
