# Supplementary material for: A VIGS screen identifies immunity in the Arabidopsis Pla‐1 accession to viruses in two different genera of the Geminiviridae
Source: Plant J. 2017 Oct 24;92(5):796–807. doi: 10.1111/tpj.13716 (PMC5725698; doi:10.1111/tpj.13716)
Supplement: Supplementary file 1 — Figure S1. Thumbnail images of CH‐42 VIGS in different Arabidopsis accessions. [file TPJ-92-796-s001.pdf]

Figure S1: Thumbnail images of *CH-42* VIGS in different Arabidopsis accessions. Most of the accessions are paired with a Col-0 plant (right) inoculated at the same time. Table S1 provides additional information.

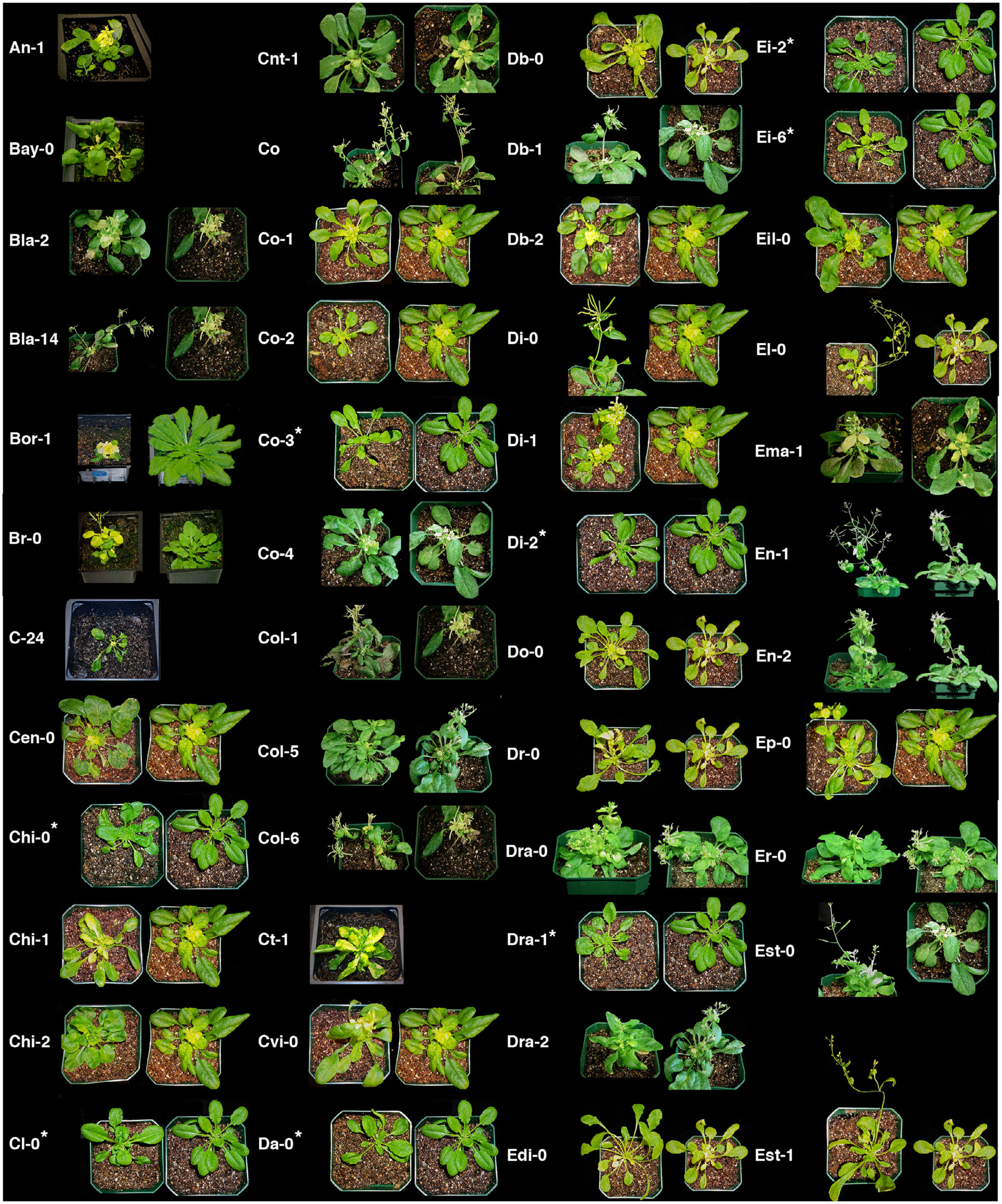

* both the accession and Col-0 were inoculated with a GFP vector to assess symptoms

** plant on the right is the same accession planted at the same time but not inoculated

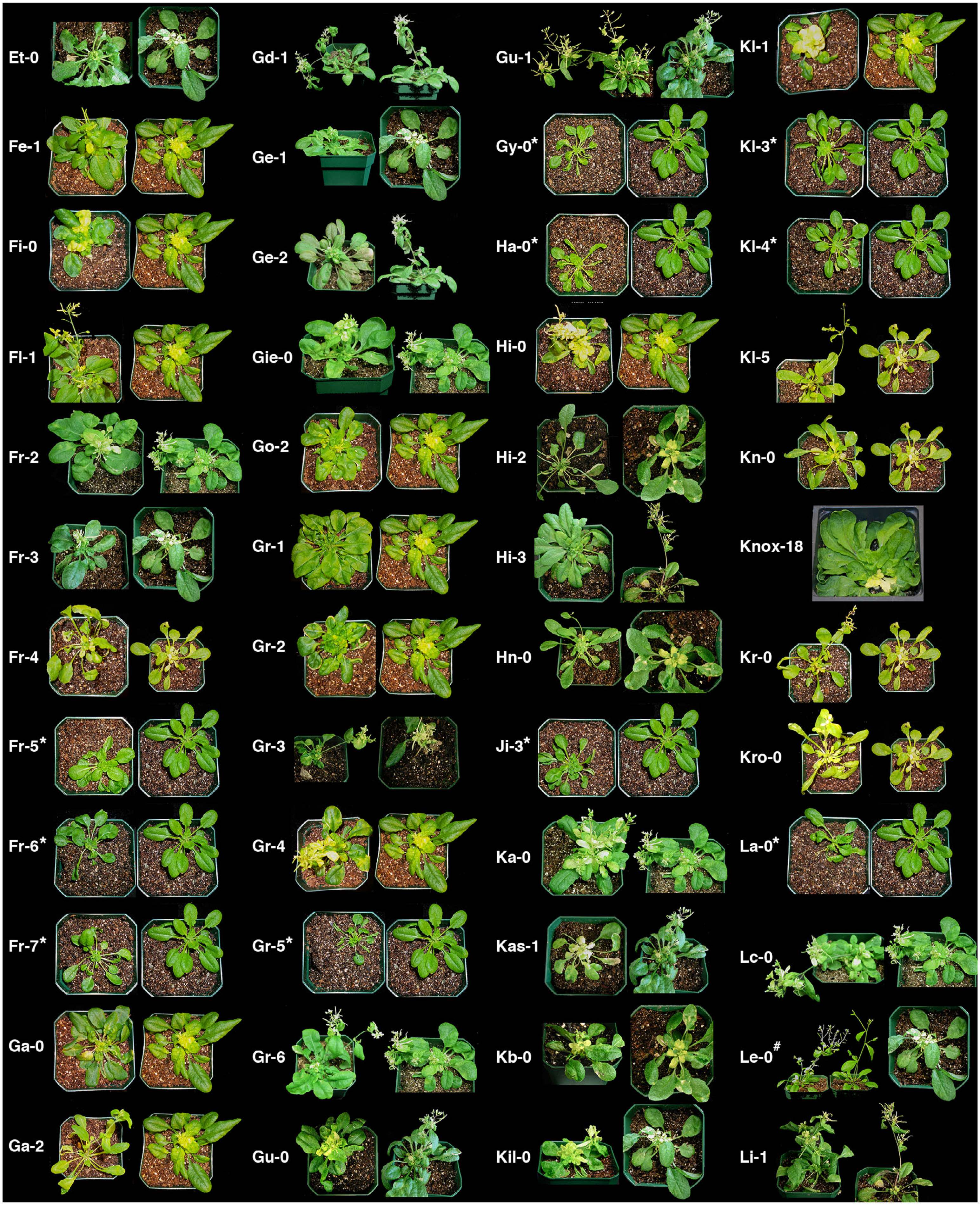

* both the accession and Col-0 were inoculated with a GFP vector to assess symptoms

** plant on the right is the same accession planted at the same time but not inoculated

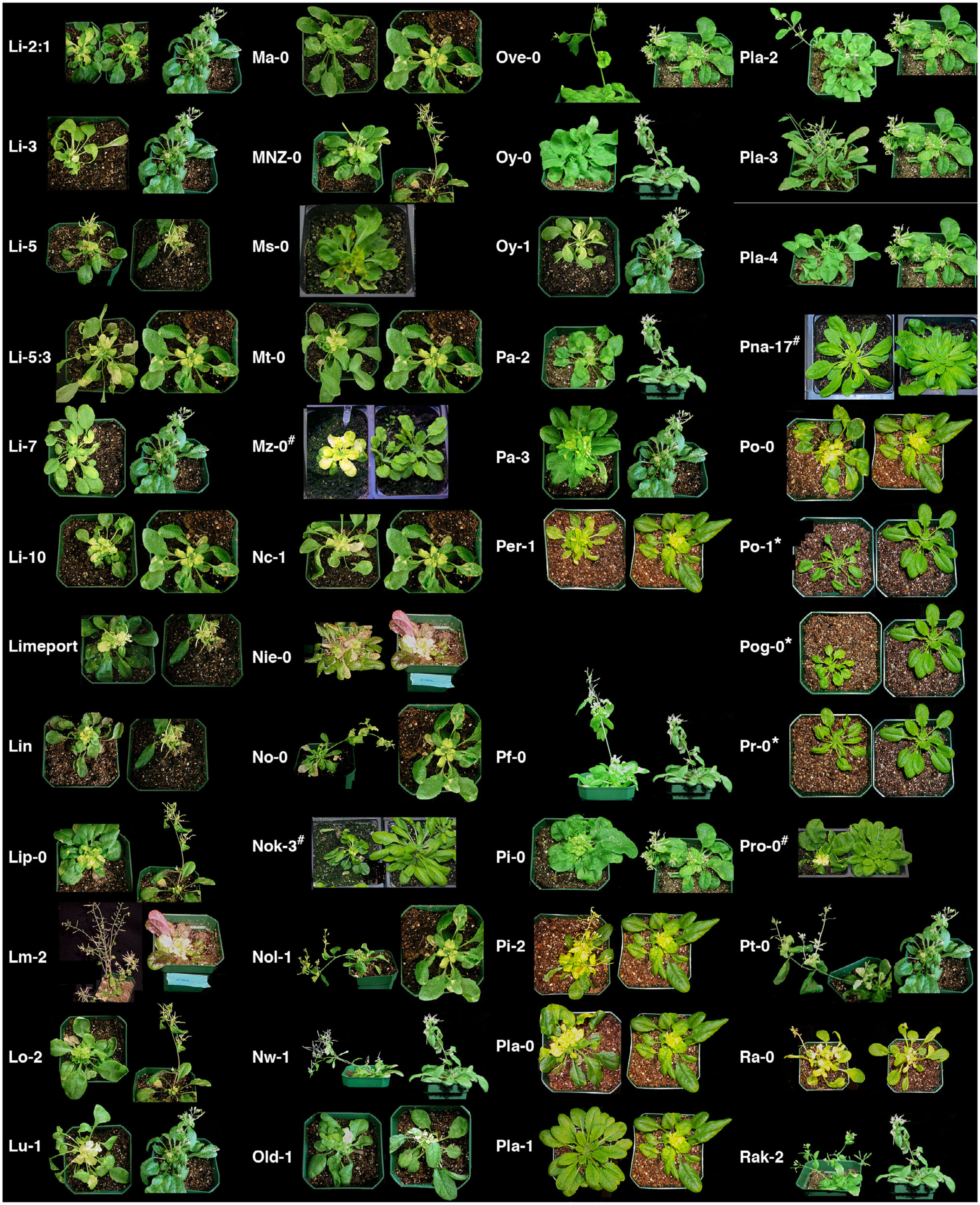

* both the accession and Col-0 were inoculated with a GFP vector to assess symptoms

** plant on the right is the same accession planted at the same time but not inoculated

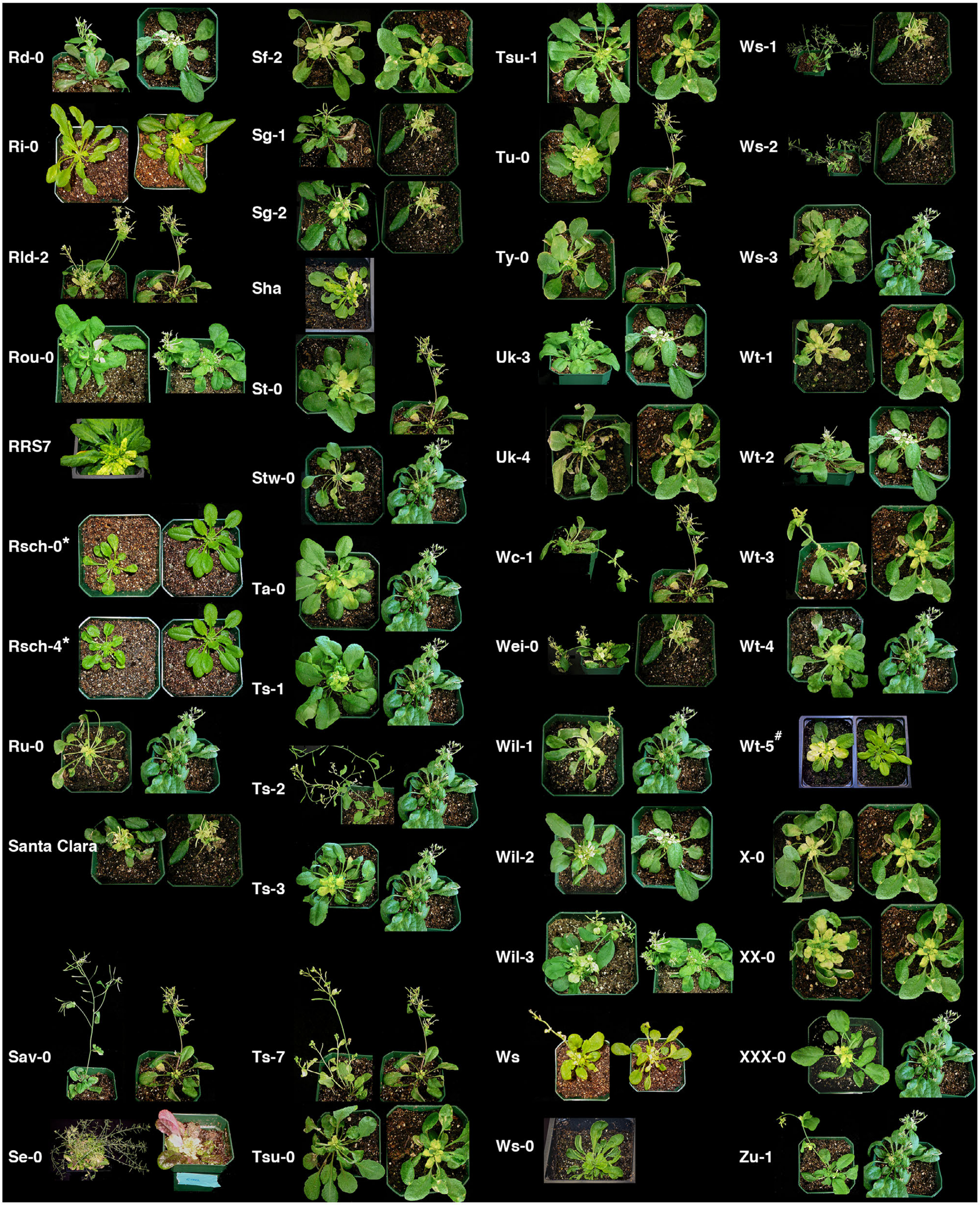

* both the accession and Col-0 were inoculated with a GFP vector to assess symptoms

** plant on the right is the same accession planted at the same time but not inoculated
