# Supplementary material for: A VIGS screen identifies immunity in the Arabidopsis Pla‐1 accession to viruses in two different genera of the Geminiviridae
Source: Plant J. 2017 Oct 24;92(5):796–807. doi: 10.1111/tpj.13716 (PMC5725698; doi:10.1111/tpj.13716)
Supplement: Supplementary file 2 — Figure S2. Examples of accessions with attenuated symptoms and increased silencing over time. [file TPJ-92-796-s002.pdf]

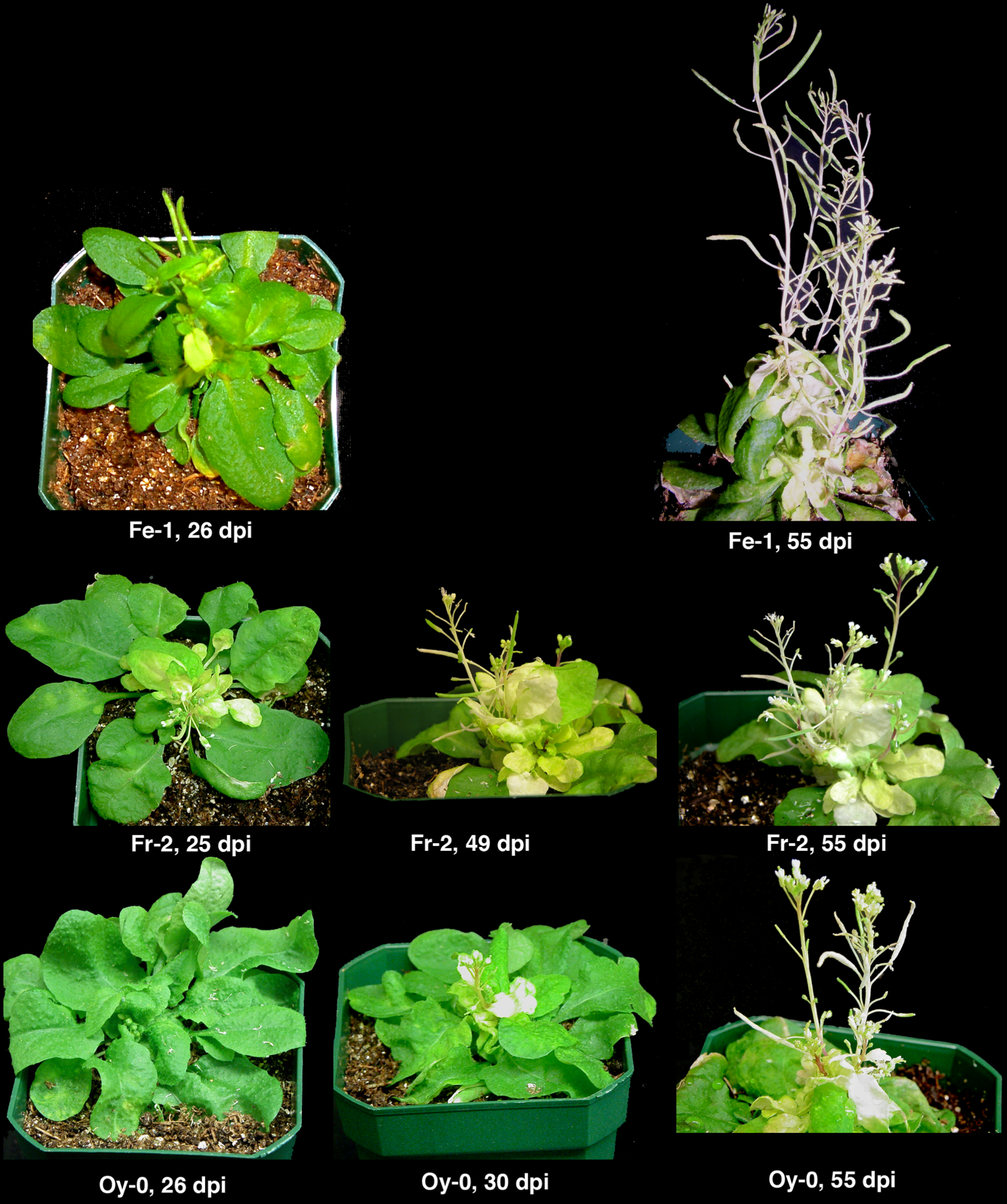

Figure S2: Examples of accessions with attenuated symptoms and increased silencing over time. Photos on the left show three accessions at the time of the screen, 25 dpi, when they were put into Class A due to symptoms and limited silencing. However photos on the left suggest they belong in Class B at 45 – 55 dpi. Oy-0 did not show VIGS until 30 dpi.
