# Supplementary material for: A VIGS screen identifies immunity in the Arabidopsis Pla‐1 accession to viruses in two different genera of the Geminiviridae
Source: Plant J. 2017 Oct 24;92(5):796–807. doi: 10.1111/tpj.13716 (PMC5725698; doi:10.1111/tpj.13716)
Supplement: Supplementary file 3 — Figure S3. CaLCuV AL1 frameshift mutation abolishes viral DNA replication in Nicotiana tabacum (NT1) protoplasts. [file TPJ-92-796-s003.pdf]

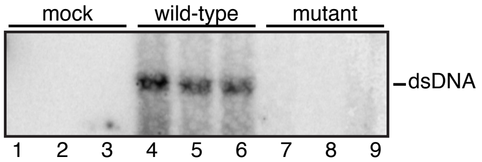

Figure S3: CaLCuV *AL1* frameshift mutation abolishes viral DNA replication in *Nicotiana tabacum* (NT1) protoplasts. Protoplasts were electroporated with no viral DNA (mock) (lanes 1-3), wild-type CaLCuV A DNA (lanes 4 to 6) or the replication-deficient CaLCuV A mutant DNA (lanes 7 to 9) in triplicate. Total DNA was extracted at 48-h post transfection and analyzed by DNA gel blotting using a ^{32}P-labeled CaLCuV DNA-A probe. dsDNA, double-stranded DNA.
