# Supplementary material for: A VIGS screen identifies immunity in the Arabidopsis Pla‐1 accession to viruses in two different genera of the Geminiviridae
Source: Plant J. 2017 Oct 24;92(5):796–807. doi: 10.1111/tpj.13716 (PMC5725698; doi:10.1111/tpj.13716)
Supplement: Supplementary file 4 — Figure S4. New growth in Pla‐1 lacks TRV:AtPDS VIGS at later time points compared with Col‐0. [file TPJ-92-796-s004.pdf]

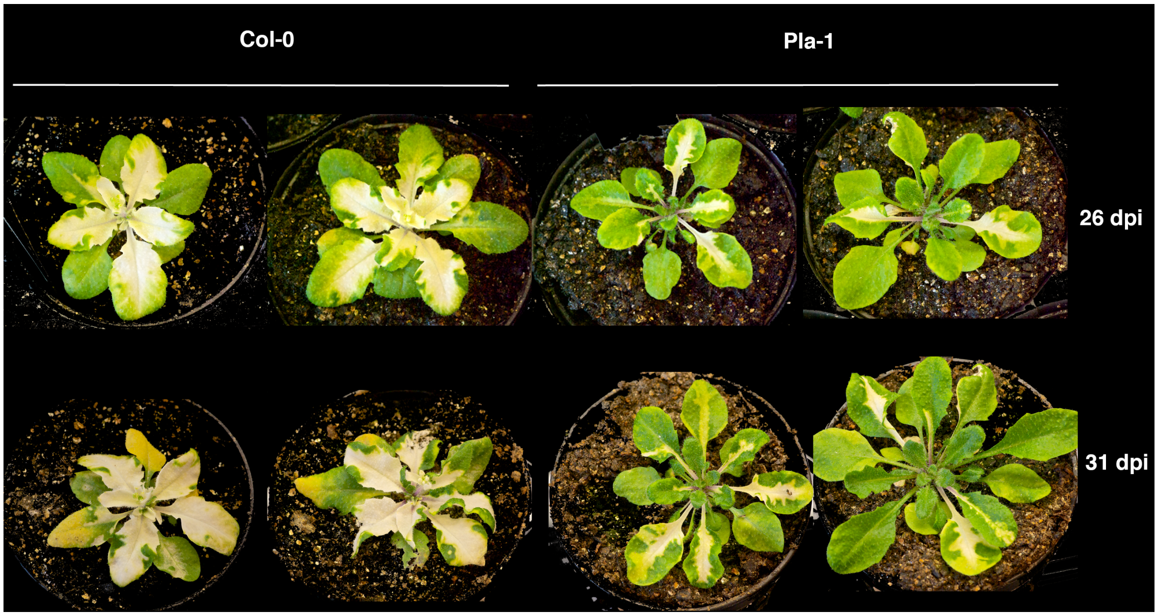

Figure S4: New growth in Pla-1 lacks TRV:*AtPDS* VIGS at later time points compared to Col-0. The same plants are shown at two different time points, 26 and 31 dpi. The 4 youngest leaves of Pla-1 plants lack visible VIGS at 31 dpi while, in Col-0, they retain silencing.
