# Supplementary material for: A VIGS screen identifies immunity in the Arabidopsis Pla‐1 accession to viruses in two different genera of the Geminiviridae
Source: Plant J. 2017 Oct 24;92(5):796–807. doi: 10.1111/tpj.13716 (PMC5725698; doi:10.1111/tpj.13716)
Supplement: Supplementary file 5 — Figure S5. Pla‐1 is susceptible to TuMV. [file TPJ-92-796-s005.pdf]

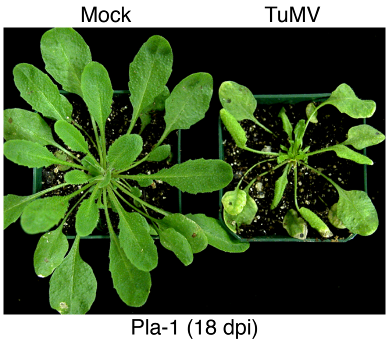


**Figure S5**: Pla-1 is susceptible to TuMV. Mock-inoculated (left panel) and TuMV-inoculated Pla-1 (right panel) photographed at 18 dpi.
