# Supplementary material for: A VIGS screen identifies immunity in the Arabidopsis Pla‐1 accession to viruses in two different genera of the Geminiviridae
Source: Plant J. 2017 Oct 24;92(5):796–807. doi: 10.1111/tpj.13716 (PMC5725698; doi:10.1111/tpj.13716)
Supplement: Supplementary file 6 — Figure S6. CaLCuV symptom score key. [file TPJ-92-796-s006.pdf]

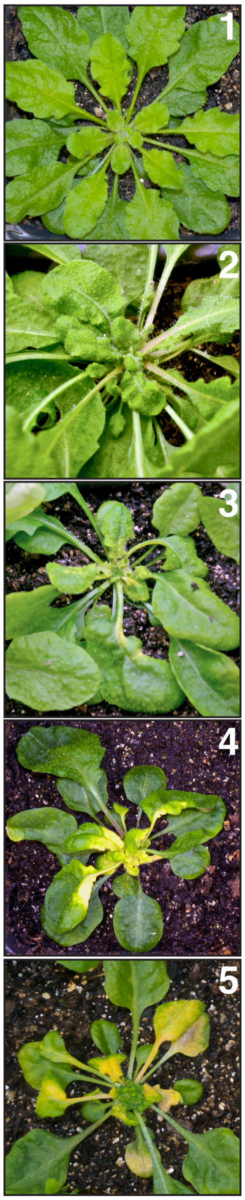

Figure S6: CaLCuV symptom score key. Photographs of F_2 plants agroinoculated with wild-type CaLCuV representing the symptom score on a scale from 1-5. 1, no symptoms; 2, curling of young leaves; 3, reticulated chlorosis in young and older leaves, leaf deformation and stunting; 4, prominent chlorosis and curling of new and older leaves, twisting of older leaves, leaf deformation and stunting; 5, severe chlorosis and stunting, new growth arrest and meristem area death.
