# Supplementary material for: A VIGS screen identifies immunity in the Arabidopsis Pla‐1 accession to viruses in two different genera of the Geminiviridae
Source: Plant J. 2017 Oct 24;92(5):796–807. doi: 10.1111/tpj.13716 (PMC5725698; doi:10.1111/tpj.13716)
Supplement: Supplementary file 7 — Figure S7. QTL maps from F2:3 families. [file TPJ-92-796-s007.pdf]

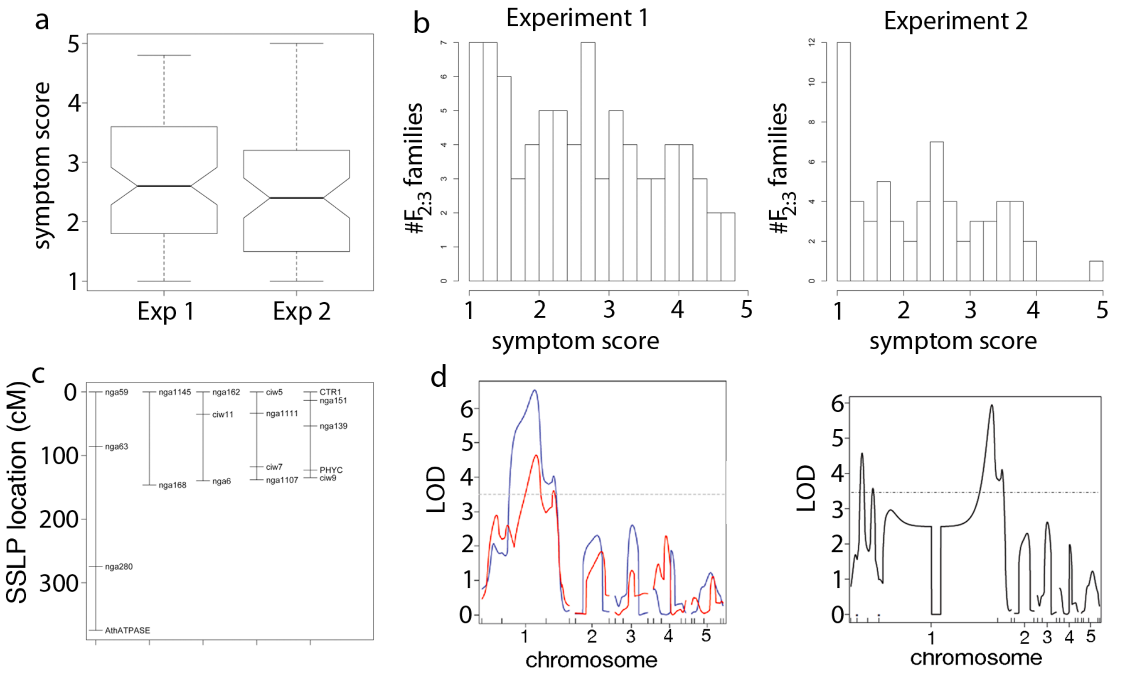

Figure S7: QTL maps from $F_{2:3}$ families. 81 families derived from a cross between Pla-1 and Col-0 were scored for symptoms in three separate experiments. A box plot (a) and histogram (b) of the responses in experiments 1 and 2 are shown to compare symptom severity and overall variation. In the histogram, the number of families is shown on the Y axis and symptom level on the X axis. The first experiment scored 14 families as resistant (average score less than 0.5) out of 81 total (chi square 2.61, P = .11) while the second had 16 out of 73 total (chi square .37, P = .54). The relative position of the SSLP markers used for QTL mapping is shown in (c). In (d), QTL maps for the first 2 experiments are shown on the left. In the third experiment (right side), 2 additional SSLP markers were added that flank nga59 and nga63; their positions are indicated by asterisks on the x axis of the QTL plot. The F19P19-75410 marker is to the left of nga59 and the AtS0392 marker is on the right of nga63.
