# Supplementary material for: A VIGS screen identifies immunity in the Arabidopsis Pla‐1 accession to viruses in two different genera of the Geminiviridae
Source: Plant J. 2017 Oct 24;92(5):796–807. doi: 10.1111/tpj.13716 (PMC5725698; doi:10.1111/tpj.13716)
Supplement: Supplementary file 12 — Methods S1. CaLCuV A DNA replication assay in Nicotiana tabacum (NT1) protoplasts. Methods S2. TuMV inoculation. Methods S3. QTL mapping using F2:3 families. Methods S4. Generation of the geminivirus immunity candidate gene list. [file TPJ-92-796-s012.docx]

**Supporting Experimental Methods**

**Methods S1: CaLCuV A DNA replication assay in *Nicotiana tabacum* (NT1) protoplasts.** Protoplasts were prepared from NT1 cells and electroporated with 10 μg of the wild-type CaLCuV A (pCPCbLCVA.003) plasmid (positive control), the replication-deficient CaLCuV A mutant plasmid (pCaLCuVA:FSAL1mut) or no viral DNA (negative control) using a published protocol (Fontes *et al.*, 1994). The transfected cells were analyzed for CaLCuV A DNA replication as previously described (Shen *et al.*, 2014).

**Methods S2: TuMV inoculation.** The TuMV inoculum consisted of freshly prepared crude sap from TuMV-infected Arabidopsis leaves. To obtain the crude sap, leaves were ground in 50 mM Phosphate Buffer, pH 7.5 (Martin Martin *et al.*, 1999). Two to three rosette leaves of five-week-old Pla-1 were dusted with carborundum and rubbed with 5-10 μL of TuMV inoculum. Symptoms were scored and plants were photographed at 18 dpi.

**Methods S3: QTL mapping using F_2:3_ families.** In the first set of mapping experiments, 83 different F_2_ plants were selfed to create F_2:3_ families and 5 F_3_ progeny from each family were agroinoculated with wild-type CaLCuV and scored for symptoms at 21 dpi. DNA from the 5 progeny was pooled and scored for the presence of 20 SSLP markers. Members of the 83 F_2:3_ families were inoculated in three different experiments and R/qtl was used to analyze the results.

**Methods S4: Generation of the geminivirus immunity candidate gene list.** Pla-1 was sequenced using the Illumina Hi-Seq platform at the Genomics Science Laboratory at NCSU. To ensure high fidelity sequence, an entire lane was devoted to Pla-1. A .sam file of the aligned sequencing data was created using BWA mem 0.7.15. We converted the .sam file to sorted .bam using samtools sam to sorted bam 0.1.19.sh. Duplicated reads were removed using samtools rmdup 0.1.19. 167,172,583 reads were properly paired. A Pla-1 SNP list was generated using samtools mpileup 0.1.19 with Col-0 (version TAIR10) as the reference genome. There are 31,245 SNPs between Col-0 and Pla-1. A list of Pla-0 Indels/Addtions/SNPs was downloaded from the 1001 genomes website (<http://tools.1001genomes.org/vcfsubset/#select_strains>). The Pla-0 list was limited to SNPs and compared to Pla-1 over the region between 8 to 13 megabases on chromosome 1 using Sequel Pro 1.1.2. SNPs duplicated in the two genomes were removed to create a new list of SNPs unique to Pla-1. Out of the 31,245 SNPs, 28,203 SNPs are shared between Pla-1 and Pla-0 and 3042 SNPs are unique Pla-1, which are potential candidate SNPs for geminivirus immunity. A list of the genes found in the same region was downloaded from TAIR (<https://gbrowse.arabidopsis.org/cgi-bin/gb2/gbrowse/arabidopsis/>) and used to eliminate intergenic SNPs. A list of candidate genes containing one or more Pla-1-specific SNPs was created using Sequel Pro 1.1.2 (Table S4). Gene descriptions were obtained from TAIR (<https://www.arabidopsis.org/tools/bulk/genes/index.jsp>).

**Supporting References**

**Fontes, E.P., Eagle, P.A., Sipe, P.S., Luckow, V.A. and Hanley-Bowdoin, L.** (1994) Interaction between a geminivirus replication protein and origin DNA is essential for viral replication. *J. Biol. Chem.* , **269** 8459–8465.

**Martin Martin, A., Cabrera y Poch, H.L., Martinez Herrera, D. and Ponz, F.** (1999) Resistances to turnip mosaic potyvirus in Arabidopsis thaliana. *Mol Plant Microbe Interact*, **12**, 1016-1021.

**Shen, W., Dallas, M.B., Goshe, M.B. and Hanley-Bowdoin, L.** (2014) SnRK1 phosphorylation of AL2 delays Cabbage leaf curl virus infection in Arabidopsis. *J Virol*, **88**, 10598-10612.
