# Supplementary material for: A VIGS screen identifies immunity in the Arabidopsis Pla‐1 accession to viruses in two different genera of the Geminiviridae
Source: Plant J. 2017 Oct 24;92(5):796–807. doi: 10.1111/tpj.13716 (PMC5725698; doi:10.1111/tpj.13716)
Supplement: Supplementary file 13 [file TPJ-92-796-s013.docx]

**Short Supporting Information Legends**

**Figure S1.** Thumbnail images of *CH-42* VIGS in different Arabidopsis accessions. Most of the accessions are paired with Col-0 plant (right) inoculated at the same time. Table S1 additional information. *Both the accession and Col-0 were inoculated with a GFP vector to assess symptoms. **Plant on the right is the same accession planted at the same time but not inoculated.

**Figure S2.** Examples of accessions with attenuated symptoms and increased silencing over time. Photos on the left show three accessions at the same time of the screen, 25 dpi, when they were put into Class A due to symptoms and limited silencing. However, photos on the left suggest they belong in Class B at 45–55 dpi. Oy-0 did not show VIGS until 30 dpi.

**Figure S3.** CaLCuV *AL1* frameshift mutation abolishes viral DNA replication in *Nicotiana tabacum* (NT1) protoplasts. Protoplasts were electroporated with no viral DNA (mock) (lanes 1-3), wild-type CaLCuV A DNA (lanes 4 to 6) or the replication deficient CaLCuV A mutant DNA (lanes 7 to 9) in triplicate. Total DNA was extracted at 48-h post transfection and analyzed by DNA gel blotting using a ^32^P-labeled CaLCuV DNA-A probe. dsDNA, double-stranded DNA.

**Figure S4.** New growth in Pla-1 lacks TRV:*AtPDS* VIGS at later time points compared to Col-0. The same plants are shown at two different time points, 26 and 31 dpi. The 4 youngest leaves of Pla-1 plants lack visible VIGS at 31 dpi while, in Col-0, they retain silencing.

**Figure S5.** Pla-1 is susceptible to TuMV. Mock-inoculated (left panel) and TuMV-inoculated Pla-1 (right panel) photographed at 18 dpi.

**Figure S6.** CaLCuV symptom score key. Photographs of F_2_ plants agroinoculated with wild-type CaLCuV representing the symptom score on a scale from 1–5. 1, no symptoms; 2, curling of young leaves; 3, reticulated chlorosis in young and older leaves, leaf deformation and stunting; 4, prominent chlorosis and curling of new and older leaves, twisting of older leaves, leaf deformation and stunting; 5, severe chlorosis and stunting, new growth arrest and meristem area death.

**Figure S7.** QTL maps from F_2:3_ families. 81 families derived from a cross between Pla-1 and Col-0 were scored for symptoms in three separate experiments. A box plot (a) and histogram (b) of the responses in experiments 1 and 2 are shown to compare symptom severity and overall variation. In the histogram, the number of families is shown on the Y axis and symptom level on the X axis. The first experiment scored 14 families as resistant (average score less than 0.5) out of 81 total (chi square 2.61, P = .11) while the second had 16 out of 73 total (chi square .37, P = .54). The relative position of the SSLP markers used for QTL mapping is shown in (c). In (d), QTL maps for the first 2 experiments are shown on the left. In the third experiment (right side), 2 additional SSLP markers were added that flank nga59 and nga63; their positions are indicated by asterisks on the x axis of the QTL plot. The F19P19-75410 marker is to the left of nga59 and the AtS0392 marker is on the right of nga63.

**Table S1.** Response of 190 Arabidopsis accessions to inoculation with the CaLCuVA:CH-42 VIGS vector or to CaLCuVA:LUC.

**Table S2.** SSLP markers for Pla-1 and Col-0.

**Table S3.** SNPs for Pla-1 and Col-0.

**Table S4.** Candidate Genes for *geminivirus immunity Pla-1-1* (*gip-1*).

**Methods S1.** CaLCuV A DNA replication assay in *Nicotiana tabacum* (NT1) protoplasts.

**Methods S2.** TuMV inoculation.

**Methods S3.** QTL mapping using F_2:3_ families.

**Methods S4.** Generation of the geminivirus immunity candidate gene list.
